# Reactions of 3′,5′-di-*O*-acetyl-2′-deoxyguansoine and 3′,5′-di-*O*-acetyl-2′-deoxyadenosine to UV light in the presence of uric acid

**DOI:** 10.1186/s41021-022-00234-5

**Published:** 2022-01-21

**Authors:** Toshinori Suzuki, Miyu Takeuchi, Atsuko Ozawa-Tamura

**Affiliations:** School of Pharmacy, Shujitsu University, 1-6-1 Nishigawara, Okayama, 703-8516 Japan

**Keywords:** Uric acid, Deoxyguanosine, Deoxyadenosine, Photosensitizer, UV light

## Abstract

**Introduction:**

Recently, it was revealed that uric acid is a photosensitizer of reactions of nucleosides on irradiation with UV light at wavelengths longer than 300 nm, and two products generated from 2′-deoxycytidine were identified. In the present study, UV reactions of acetylated derivatives of 2′-deoxyguansoine and 2′-deoxyadenosine were conducted and their products were identified.

**Findings:**

Each reaction of 3′,5′-di-*O*-acetyl-2′-deoxyguansoine or 3′,5′-di-*O*-acetyl-2′-deoxyadenosine with UV light at wavelengths longer than 300 nm in the presence of uric acid generated several products. The products were separated by HPLC and identified by comparing UV and MS spectra of the products with previously reported values. The major products were spiroiminodihydantoin, imidazolone, and dehydro-iminoallantoin nucleosides for 3′,5′-di-*O*-acetyl-2′-deoxyguansoine, and an adenine base and a formamidopyrimidine nucleoside for 3′,5′-di-*O*-acetyl-2′-deoxyadenosine.

**Conclusions:**

If these damages caused by uric acid with sunlight occur in DNA of skin cells, mutations may arise. We should pay attention to the genotoxicity of uric acid in terms of DNA damage to dGuo and dAdo sites mediated by sunlight.

## Introduction

Since uric acid is the final metabolic product of purine catabolism in humans, it exists ubiquitously in various cells and body fluids at relatively high concentrations [1, 2]. Uric acid is an important antioxidant in humans [3]. However, it can also act as a pro-oxidant inducing oxidative stress of cells [4, 5]. It has been reported that uric acid in cultured mouse skin cells is increased by UV irradiation, and that uric acid on the human skin surface is increased by sunlight exposure [6, 7]. An epidemiological cancer study reported that the incidence of non-melanoma skin cancer showed a positive association with the serum uric acid concentration [8]. Recently, we showed that uric acid is a photosensitizer of reactions of nucleosides on irradiation with UV light at wavelengths longer than 300 nm [9]. The reactions of nucleosides were suppressed by radical scavengers. Two products from 2′-deoxycytidine (dCyd) were separated by reversed phase (RP) HPLC and identified by MS and NMR as *N*^4^-hydroxy-2′-deoxycytidine and *N*^4^,5-cyclic amide-2′-deoxycytidine, formed by cycloaddition of an amide group from uric acid. The results using ^15^N-labeled uric acid indicated that the amide group added to dCyd originates from both the five-membered imidazole ring and six-membered pyrimidine ring of uric acid, suggesting that an unidentified radical derived from uric acid with a delocalized unpaired electron is generated. To obtain information about reaction products of nucleosides other than dCyd, we analyzed the reaction solutions of 2′-deoxyguanosine (dGuo) and 2′-deoxyadenosine (dAdo) irradiated with UV light in the presence of uric acid. However, we failed to obtain product peaks with a good resolution on RP-HPLC. Thus, acetylated derivatives of dGuo (3′,5′-di-*O*-acetyl-2′-deoxyguanosine; AcdGuo) and dAdo (3′,5′-di-*O*-acetyl-2′-deoxyadenosine; AcdAdo) were prepared and used for analysis of the UV irradiation reaction to improve the retention and separation of the products by RP-HPLC. In the present study, we show identification and quantification of the products from AcdGuo and AcdAdo by UV irradiation in the presence of uric acid.

## Materials and methods

### Materials

dGuo, dAdo, and uric acid were purchased from Sigma-Aldrich (MO, USA). Other chemicals were obtained from Sigma-Aldrich, Nacalai Tesque (Kyoto, Japan), and Tokyo Chemical Industry (Tokyo, Japan). Water was purified with a Millipore Milli-Q deionizer (MA, USA). AcdGuo and AcdAdo were synthesized from dGuo and dAdo, respectively, by acetylation using acetic anhydride as previously described [10]. AcdGuo and AcdAdo were purified by RP-HPLC.

### Irradiation conditions

For UV light irradiation, UV light originating from a 250-W high-pressure mercury lamp (SP9-250UB, Ushio, Tokyo, Japan) with an optical filter through a light guide was used to directly irradiate the surface of a solution (1 mL) in a glass vial (12 mm i.d.) without a cap at 37 °C. Longpass filter LU0300 (cut-on 300 nm) (Asahi Spectra, Tokyo, Japan) was used as the optical filter. The intensity of radiation on the surface of the sample solution was measured with a photometer (UIT-150, Ushio, Tokyo, Japan) equipped with a sensor, UVD-S254 or UVD-S365. The intensities of the UV light were 0 mW/cm^2^ for 254 nm and 264 mW/cm^2^ for 365 nm.

### HPLC and MS conditions

The HPLC system consisted of LC-10ADvp pumps and an SPD-M10Avp UV-Vis photodiode-array detector (Shimadzu, Kyoto, Japan). For the RP-HPLC, an Inertsil ODS-3 octadecylsilane column of size 4.6 × 250 mm and particle size of 5 μm (GL Sciences, Tokyo, Japan) was used. The eluent was 20 mM ammonium acetate (pH 7.0) containing acetonitrile. For AcdGuo, the acetonitrile concentration was increased from 0 to 30% over 45 min in linear gradient mode. For AcdAdo, the acetonitrile concentration was increased from 0 to 37.5% over 45 min in linear gradient mode. The column temperature was 40 °C and flow rate was 1 mL/min. The RP-HPLC chromatogram was detected at 200–500 nm. ESI-TOF/MS measurements were performed on a MicrOTOF spectrometer (Bruker, Bremen, Germany) in negative mode. The sample isolated by RP-HPLC was directly infused into the MS system by a syringe pump without a column.

### Quantitative procedures

The concentrations of the products were evaluated according to integrated peak areas on RP-HPLC chromatograms detected at 245 nm and the *ε*_245 nm_ value of each product, compared with the peak area of the standard solution of AcdGuo for the AcdGuo reactions or Ade for the AcdAdo reactions. The *ε*_245 nm_ values were used as 12,400 M^− 1^ cm^− 1^ for AcdGuo and 8450 M^− 1^ cm^− 1^ for Ade. The used *ε*_245 nm_ values of the products are indicated in Tables 1 and 2.

**Table 1:** Characteristics of Products Formed by UV Irradiation of AcdGuo with Uric Acid

Products	*t*_R_ (min)	*λ*_max_ (nm)	*m/z* (negative)	*ε*_245 nm_ (M^−1^ cm^− 1^)^a^
**1**. AcdSph (fast)	25.2	230 (shoulder)	382	5480
**2**. AcdSph (slow)	25.6	230 (shoulder)	382	5480
**3**. AcdOz	28.7	232	329	6000
**4**. AcdIz	30.9	254, 320	311	20,500
**5**. AcdIa^ox^	33.3	236	354	12,840
**6**. 8-oxo-AcdGuo	37.6	254, 295	366	15,560

^a^The values of *ε*_245 nm_ are those previously reported [10]

**Table 2:** Characteristics of Products Formed by UV Irradiation of AcdAdo with Uric Acid

Products	*t*_R_ (min)	*λ*_max_ (nm)	*m/z* (negative)	*ε*_245 nm_ (M^−1^ cm^−1^)^a^
**7**. Ade	16.5	260	134	8450
**8**. Fapy-AcdAdo (fast)	28.7	259	352	2860
**9**. Fapy-AcdAdo (slow)	29.3	259	352	2860
**10**. 5′,8-cyclo-AcdAdo (fast)	33.9	274	332	8930
**11**. 5′,8-cyclo-AcdAdo (slow)	34.6	274	332	6910
**12**. 5′-deoxy-5′,8-cyclo -AcdAdo	35.3	264	274	9560
**13**. 8-oxo-AcdAdo	38.6	212, 269	350	10,220

^a^The values of *ε*_245 nm_ were calculated from the reported *ε* values for the products of dAdo at *λ*_max_ and their UV spectra obtained in the present study [11]

## Results and discussion

### Reaction of AcdGuo

**Fig. 1:**
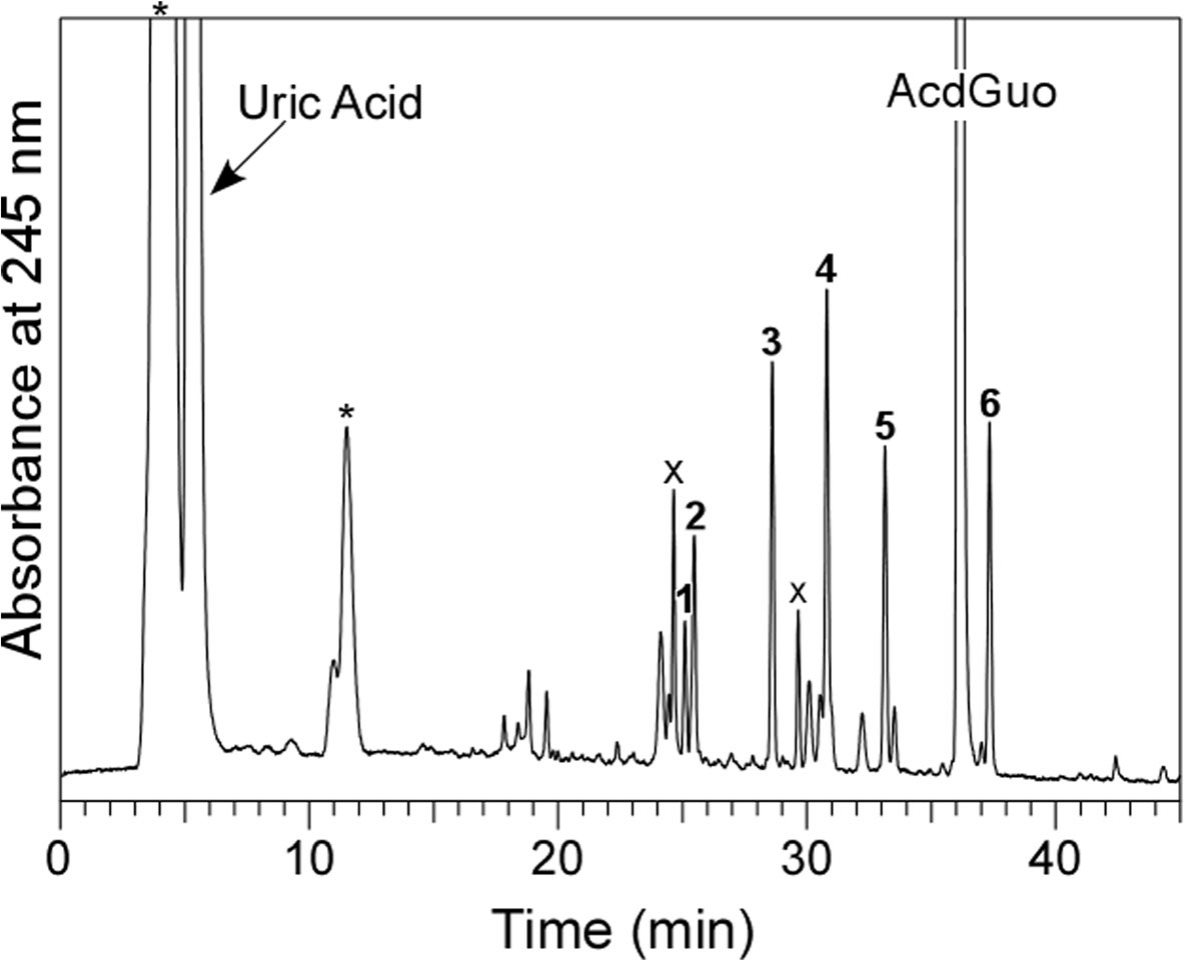
RP-HPLC chromatogram of a reaction mixture of AcdGuo with uric acid detected at 245 nm. A solution of 100 μM AcdGuo and 400 μM uric acid was irradiated with UV through a 300-nm longpass filter in 100 mM potassium phosphate buffer at pH 7.4 and 37 °C for 10 min. The HPLC system consisted of LC-10ADvp pumps and an SPD-M10Avp UV-Vis photodiode-array detector (Shimadzu, Kyoto, Japan). For RP-HPLC, an Inertsil ODS-3 octadecylsilane column of size 4.6 × 250 mm and particle size of 5 μm (GL Sciences, Tokyo, Japan) was used. The eluent was 20 mM ammonium acetate (pH 7.0) containing acetonitrile. The acetonitrile concentration was increased from 0 to 30% over 45 min in linear gradient mode. The column temperature was 40 °C and flow rate was 1 mL/min

**Fig. 2:**
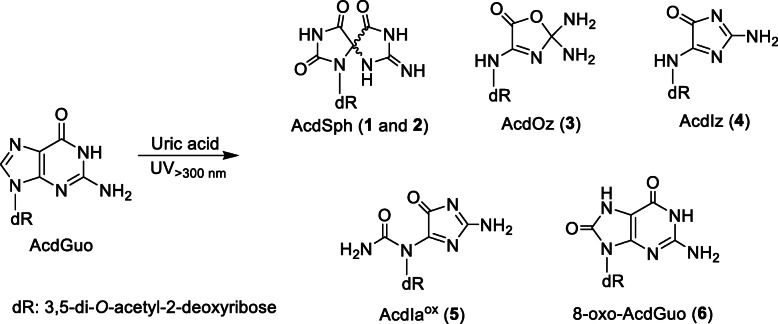
The reaction products from AcdGuo by UV irradiation in the presence of uric acid

A solution of 100 μM AcdGuo with 400 μM uric acid in 100 mM potassium phosphate buffer at pH 7.4 was irradiated with UV light from a high-pressure mercury lamp through a 300-nm longpass filter at a temperature of 37 °C for 10 min. The reaction mixture was analyzed by RP-HPLC equipped with a UV-Vis photodiode-array detector. As shown in Fig. 1, several product peaks appeared in addition to uric acid and its decomposition products, denoted by asterisks, and AcdGuo and its contaminants, denoted by crosses. Six products (Products **1**–**6**) were isolated by RP-HPLC and subjected to MS analysis. The products were identified on the basis of the similarity of their UV and MS spectra with reported values using a reaction system of AcdGuo with hypobromous acid [12]. Table 1 summarizes the characteristics of Products **1**–**6**. Products **1** and **2** were identified as diastereomers of a 3′,5′-di-*O*-acetyl derivative of spiroiminodihydantoin deoxyribonucleoside (AcdSph). Product **3** was a 3′,5′-di-*O*-acetyl derivative of diamino-oxazolone deoxyribonucleoside (AcdOz). Product **4** was a 3′,5′-di-*O*-acetyl derivative of amino-imidazolone deoxyribonucleoside (AcdIz). Product **5** was a 3′,5′-di-*O*-acetyl derivative of dehydro-iminoallantoin deoxyribonucleoside (AcdIa^ox^). Product **6** was a 3′,5′-di-*O*-acetyl derivative of 7,8-dihydro-8-oxo-2′-deoxyguanosine (8-oxo-AcdGuo). Authentic guanine (Gua) was eluted with the RP-HPLC retention time of 12.0 min as a broadened peak. The concentration of Gua in the present reaction solution could not determined due to overlapping of a peak of a decomposition product of uric acid. The structures of the reaction products from AcdGuo are shown in Fig. 2.

**Fig. 3:**
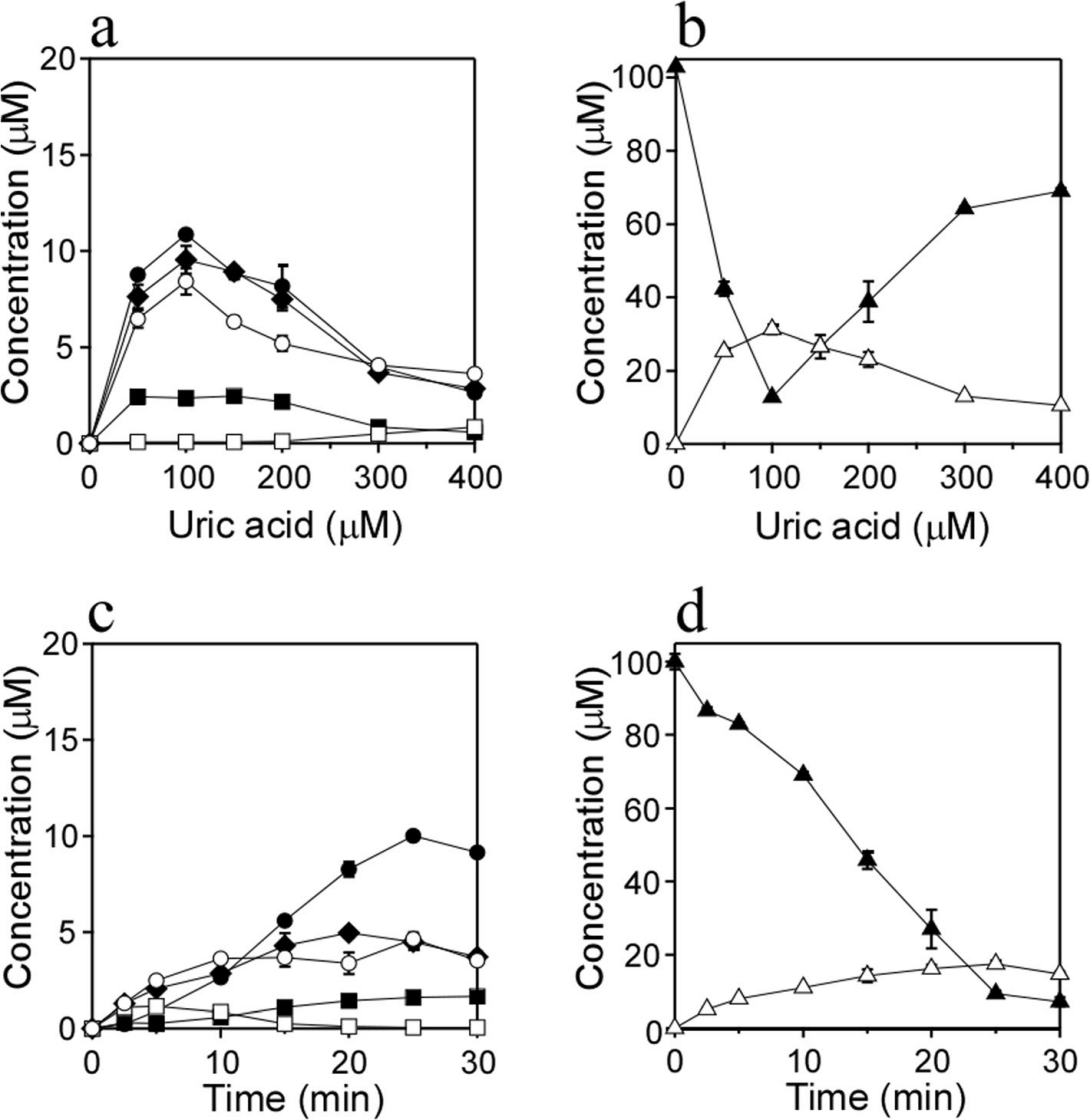
Uric acid dose-dependence and time-course of the concentration changes of AcdGuo reaction products. Uric acid dose-dependence of the concentration changes of (**a**) each product and (**b**) AcdGuo and total products, when a solution of 100 μM AcdGuo with 0–400 μM uric acid was irradiated with UV light through a 300-nm longpass filter for 10 min at pH 7.4 and 37 °C. Time-course of the concentration changes of (**c**) each product and (**d**) AcdGuo and total products, when a solution of 100 μM AcdGuo with 400 μM uric acid was irradiated with UV light through a 300-nm longpass filter for 0–30 min at pH 7.4 and 37 °C. AcdSph (**1** and **2**) (closed circle), AcdOz (**3**) (closed square), AcdIz (**4**) (closed rhombus), AcdIa^ox^ (**5**) (open circle), 8-oxo-AcdGuo (**6**) (open square), AcdGuo (closed triangle), and the total concentration of Products **1**–**6** (open triangle). All reaction mixtures were analyzed by RP-HPLC. Means ± standard deviation (S.D.) (*n* = 3) are presented

Uric acid dose-dependent changes in the reaction of AcdGuo with UV light were examined. A solution of 100 μM AcdGuo with 0–400 μM uric acid in 100 mM potassium phosphate buffer at pH 7.4 was irradiated with UV light from a high-pressure mercury lamp through a 300-nm longpass filter at a temperature of 37 °C for 10 min. The product concentrations were determined from the absorbance area of HPLC detected at 245 nm using their reported molar extinction coefficients [12]. Figure 3A shows the changes in concentrations of the products. At 0 μM uric acid, no product was detected. At around 100 μM uric acid, concentrations of all products other than 8-oxo-AcdGuo were maximal, while the concentration of 8-oxo-AcdGuo increased with an increasing uric acid concentration up to 400 μM. Over the uric acid concentration range examined, the major products were AcdSph, AcdIz, and AcdIa^ox^ with comparable yields. Figure 3B shows the AcdGuo concentration and total concentration of all six products. At 0 μM uric acid, no consumption of AcdGuo was observed. At 100 μM uric acid, the consumption of AcdGuo and total yield of the products were maximal. Over the uric acid concentration range examined, the total yield of all products was approximately one-third of the consumption of AcdGuo, suggesting that further reactions of the products or other reactions without these products occur. Irradiation time-dependent changes in the reaction of AcdGuo with UV light were examined. A solution of 100 μM AcdGuo with 400 μM uric acid in 100 mM potassium phosphate buffer at pH 7.4 was irradiated with UV light at a temperature of 37 °C for 0–30 min. Figure 3C shows the changes in concentrations of the products. When the solution was incubation at 37 °C for 10 min without UV irradiation, no product was detected. At 5 min UV irradiation, the major products were AcdIz and AcdIa^ox^. At 15–30 min, the main product was AcdSph. The concentration of 8-oxo-AcdGuo was maximal at 5 min, then decreased, with an intermediate kinetics profile. It has been reported that spirohydantoin nucleoside (dSph) is generated as a two-step oxidation product of dGuo via 8-oxo-dGuo [13]. In the present system, AcdSph should also lead to further oxidation of 8-oxo-AcdGuo. Reportedly AcdIa^ox^ is generated as a three-step oxidation product of dGuo [14]. Since AcdIa^ox^ was one of the major products in the present reaction, the reaction rates of these three-steps of oxidation should be relatively high. On the other hand, imidazolone nucleoside (dIz) is generated by oxidation of dGuo and subsequent degradations without 7,8-dihydro-8-oxo-2′-deoxyguanosine (8-oxo-dGuo) [15]. dIz is not stable, converting to stable oxazolone nucleoside (dOz) with a half-life of 2.5 h at 37 °C in a neutral solution at pH 7 [16]. The present results showing a gradual increase in the AcdOz concentration with an increase in the irradiation time and decrease of AcdIz at 20–30 min would be explainable by the instability of AcdIz. Figure 3D shows the AcdGuo concentration and total concentration of all products. The consumption of AcdGuo increased in a time-dependent manner. Although the total concentration of products increased with an increasing irradiation time, the change at 15–30 min was slight, suggesting that further reactions occur involving the products.

Mutations caused by the sites of some of these products generated in DNA have been reported as follows: dSph in an oligonucleotide strongly blocks nucleotide incorporation by DNA polymerases, and causes both G to T and G to C transversion mutations when duplication occurs over this lesion [17]. In vitro nucleotide insertion by Klenow fragment exo^−^ opposite dOz in an oligonucleoside induces mainly dAMP incorporation, suggesting that the formation of dOz in DNA may cause G to T transversion [18]. For 8-oxo-dGuo, dCMP and dAMP are incorporated opposite 8-oxo-dGuo in an oligonucleotide by DNA polymerases [11]. When dAMP is incorporated, G to T transversion mutation occurs.

### Reaction of AcdAdo

**Fig. 4:**
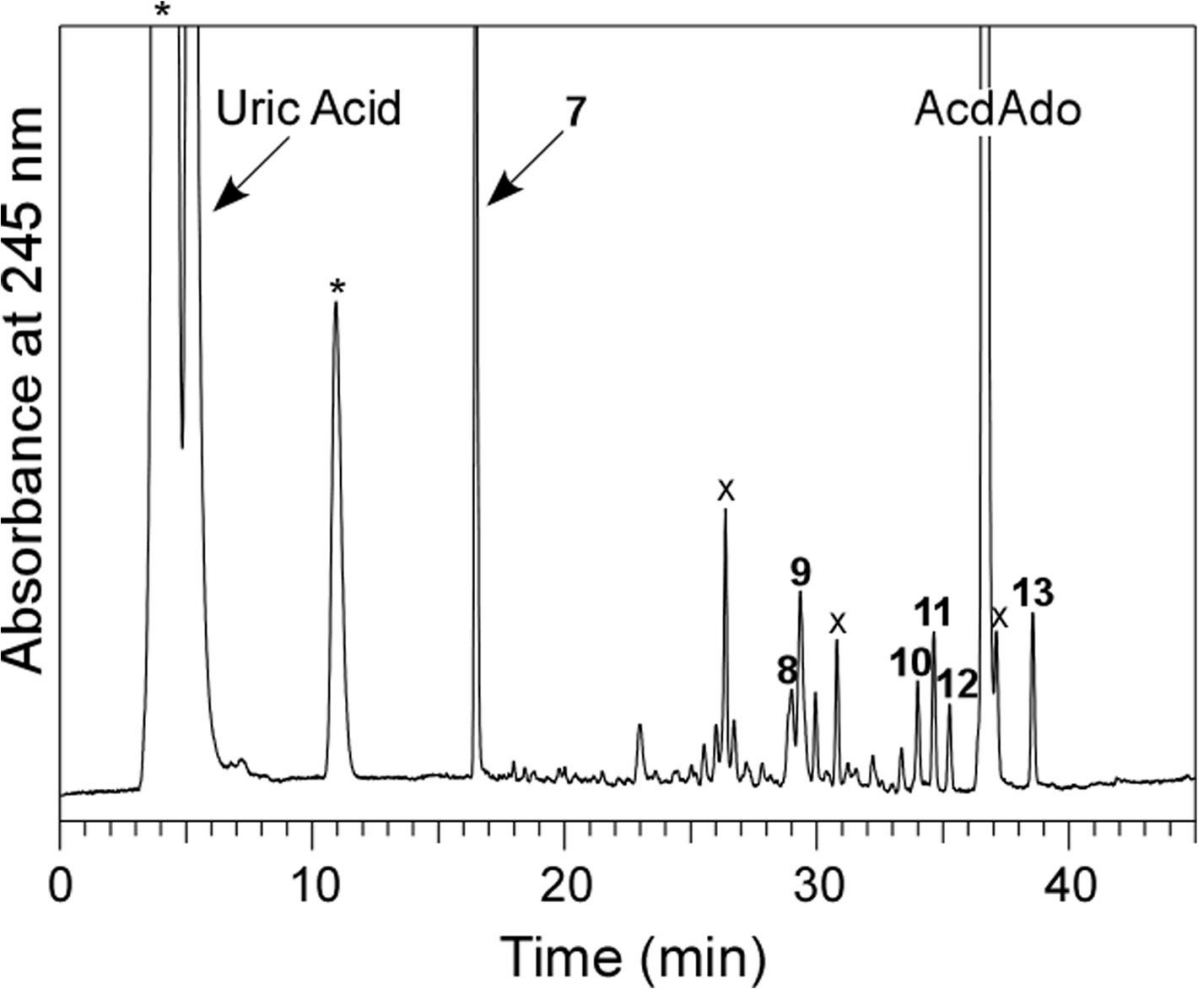
RP-HPLC chromatogram of a reaction mixture of AcdAdo with uric acid detected at 245 nm. A solution of 100 μM AcdAdo and 400 μM uric acid was irradiated with UV through a 300-nm longpass filter in 100 mM potassium phosphate buffer at pH 7.4 and 37 °C for 10 min. The HPLC conditions were the same as shown in Fig. 1 excluding the acetonitrile concentration. The acetonitrile concentration was increased from 0 to 37.5% over 45 min in linear gradient mode

**Fig. 5:**
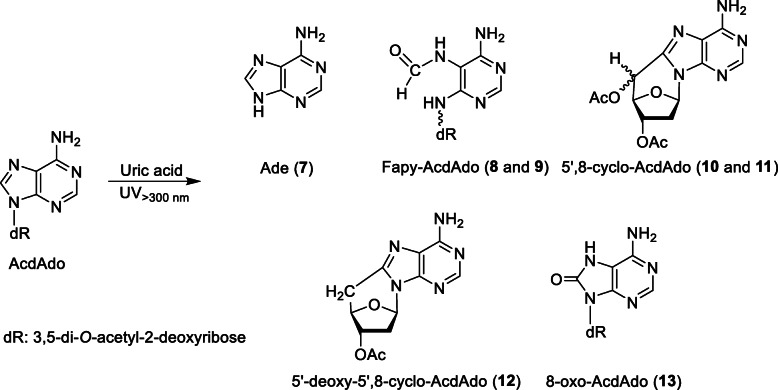
The reaction products from AcdAdo by UV irradiation in the presence of uric acid

A solution of 100 μM AcdAdo with 400 μM uric acid in 100 mM potassium phosphate buffer at pH 7.4 was irradiated with UV light through a 300-nm longpass filter at a temperature of 37 °C for 10 min. The reaction mixture was analyzed by RP-HPLC. As shown in Fig. 4, several product peaks appeared in addition to uric acid and its decomposition products, denoted by asterisks, and AcdAdo and its contaminants, denoted by crosses. Seven products (Products **7**–**13**) were isolated by RP-HPLC and subjected to MS analysis. The products were identified on the basis of coincidence of their UV and MS spectra with corresponding reported values using a reaction system of dAdo with the Fenton system [19]. Table 2 summarizes the characteristics of Products **7**–**13**. Product **7** was identified as adenine (Ade). Products **8** and **9** were diastereomers of a 3′,5′-di-*O*-acetyl derivative of formamidopyrimidine deoxyribonucleoside (Fapy-AcdAdo). Products **10** and **11** were diastereomers of a 3′,5′-di-*O*-acetyl derivative of 5′,8-cyclo-2′-deoxyadenosine (5′,8-cyclo-AcdAdo). Product **12** was a 3′-*O*-acetyl derivative of 5′-deoxy-5′,8-cyclo-2′-deoxyadenosine (5′-deoxy-5′,8-cyclo-AcdAdo). Product **13** was a 3′,5′-di-*O*-acetyl derivative of 7,8-dihydro-8-oxo-2′-deoxyadenosine (8-oxo-AcdAdo). The structures of the reaction products from AcdAdo are shown in Fig. 5.

**Fig. 6:**
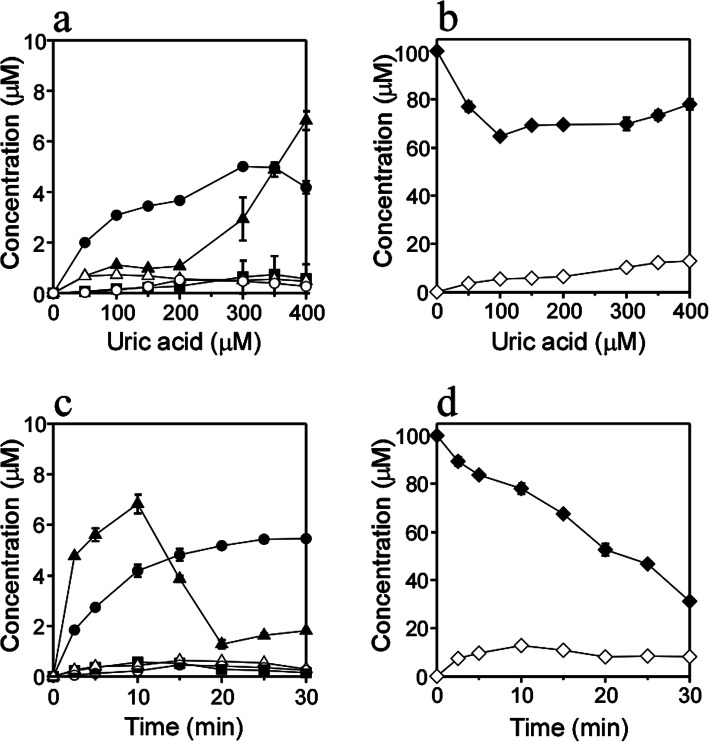
Uric acid dose-dependence and time-course of the concentration changes of AcdAdo reaction products. Uric acid dose-dependence of the concentration changes of (**a**) each product and (**b**) AcdAdo and total products, when a solution of 100 μM AcdAdo with 0–400 μM uric acid was irradiated with UV light through a 300-nm longpass filter for 10 min at pH 7.4 and 37 °C. Time-course of the concentration changes of (**c**) each product and (**d**) AcdAdo and total products, when a solution of 100 μM AcdAdo with 400 μM uric acid was irradiated with UV light through a 300-nm longpass filter for 0–30 min at pH 7.4 and 37 °C. Ade (**7**) (closed circle), Fapy-AcdAdo (**8** and **9**) (closed triangle), 5′,8-cyclo-AcdAdo (**10** and **11**) (closed square), 5′-deoxy-5′,8-cyclo-AcdAdo (**12**) (open circle), 8-oxo-AcdAdo (open triangle), AcdAdo (closed rhombus), total concentration of Products **7**–**13** (open rhombus). All the reaction mixtures were analyzed by RP-HPLC. Means ± standard deviation (S.D.) (*n* = 3) are presented

Uric acid dose-dependent changes in the reaction of AcdAdo with UV light were examined. A solution of 100 μM AcdAdo with 0–400 μM uric acid in 100 mM potassium phosphate buffer at pH 7.4 was irradiated with UV light at a temperature of 37 °C for 10 min. The product concentrations were determined from the absorbance area of HPLC detected at 245 nm using their molar extinction coefficients, which were calculated from the reported values at *λ*_max_ for corresponding products of dAdo and UV spectra of the products of AcdAdo obtained in the present study [19]. Figure 6A shows the changes in concentrations of the products. At 0 μM uric acid, no product was detected. At 5–300 μM uric acid, the main product was Ade. At above 300 μM uric acid, the concentration of Fapy-AcdAdo markedly increased. At 400 μM uric acid, the main product was Fapy-AcdAdo. The concentration of 8-oxo-AcdAdo was almost constant at 50–400 μM uric acid. Reportedly, formamidopyrimidine deoxyribonucleoside (Fapy-dAdo) and 7,8-dihydro-8-oxo-2′-deoxyadenosine (8-oxo-dAdo) are generated from a common radical intermediate formed from dAdo by oxidative stress via subsequent reduction and oxidation, respectively [20, 21]. In the present study, the reduction reaction generating Fapy-AcdAdo may become dominant in the presence of a higher concentration of uric acid. Figure 6B shows the AcdAdo concentration and total concentration of Products **7**–**13**. At 0 μM uric acid, no consumption of AcdAdo was observed. The consumption of AcdAdo increased up to 100 μM uric acid, and then decreased moderately. The total generation of the products was approximately one-seventh of the consumption of AcdAdo at 100 μM uric acid and one-half at 400 μM uric acid, suggesting that further reactions involving the products or other reactions without these products occur, especially at around 100 μM uric acid. Irradiation time-dependent changes in the reaction of AcdAdo with UV light were examined. A solution of 100 μM AcdAdo with 400 μM uric acid in 100 mM potassium phosphate buffer at pH 7.4 was irradiated with UV light at a temperature of 37 °C for 0–30 min. Figure 6C shows the changes in concentrations of the products. When the solution was incubation at 37 °C for 10 min without UV irradiation, no product was detected. At up to 10 min UV irradiation, the main product was Fapy-AcdAdo. At 15–30 min, the concentration of Fapy-AcdAdo markedly decreased, suggesting that further reactions occur involving Fapy-AcdAdo. At 15–30 min, the main product was Ade. Figure 6D shows the AcdAdo concentration and total concentration of Products **7**–**13**. The consumption of AcdAdo increased in a time-dependent manner, although the total generation of the products was maximal at 10 min and decreased gradually up to 30 min.

Mutations caused by the sites of some of these products generated in DNA have been reported as follows: In vitro nucleotide insertion by the Klenow fragment exo^−^ opposite Fapy-dAdo in an oligonucleoside induces mainly dTMP incorporation, although the frequency is one-fourth that of the native dAdo in the template [21]. The frequency of misincorporation of dAMP and dGMP opposite Fapy-dAdo was 50% greater than that opposite native dAdo, suggesting increasing rates of A to T and A to C transversion mutation. For 8-oxo-dAdo, human DNA polymerase η proficiently incorporated dGMP opposite 8-oxo-dAdo, suggesting an increase of A to C transversion mutation [22]. The release of Ade base from DNA is caused via abstraction of various hydrogen atoms of deoxyribose by radicals with or without a single strand break [23, 24]. In the absence of a single strand break, an abasic site is generated in DNA. In vitro DNA synthesis by human DNA polymerase ε is strongly blocked at the abasic site analog [25]. In living cells, various mutations are induced by abasic sites [26, 27].

### Reaction mechanism

Photosensitization includes many different prosesses such as energy transfer, electron transfer, hydrogen atom abstraction, singet oxygen formation, and radical formation [28, 29]. Recently we showed that uric acid is a photosensitzer on the reaction of nucleosides, dCyd, dGuo, dAdo, and thymidine, by UV light with wavelengths longer than 300 nm [9]. These reactions were inhibited by the addition of radical scavengers, ethanol and sodium azide. For the reaction of dCyd, *N*^4^,5-cyclic amide-2′-deoxycytidine was formed by cycloaddition of an amide group from uric acid. When a ^15^N-labeled uric acid, having two ^14^N and two ^15^N atoms in the molecule, was used, *N*^4^,5-cyclic amide-2′-deoxycytidine containing both ^14^N and ^15^N atoms was generated. Singlet oxygen, hydroxyl radical, peroxynitrous acid, hypochlorous acid, and hypobromous acid did not generate *N*^4^,5-cyclic amide-2′-deoxycytidine in the presence of uric acid. These results suggest that an unidentified radical derived from uric acid with a delocalized unpaired electron is generated. All the identified products formed from acetylated dGuo and dAdo in the present UV irradiation study had already been reported in the reaction with reactive free radicals and oxidants [12, 19, 29]. It has also been reported that hydrogen atom abstraction on the sugar moiety of nucleosides induces release of the base and crosslinking between the sugar and the base, and that it on the base moiety of nucleosides induces various products having modified bases [29]. A possible reaction mechanism for the present UV reaction of AcdGuo and AcdAdo with uric acid is as follows: The radical derived from uric acid by UV irradiation induces hydrogen atom abstraction from AcdGuo and AcdAdo. When hydrogen atom abstraction from the deoxyribose moiety of AcdAdo occurs, Ade, 5′,8-cyclo-AcdAdo, and 5′-deoxy-5′,8-cyclo-AcdAdo are generated. On the other hand, when hydrogen atom is abstracted from the base moieties of AcdGuo and AcdAdo, the other products are generated. Further studies are needed to reveal the detailed reaction mechanism.

## Conclusions

The present study showed that in the presence of uric acid, a photosensitizer, AcdGuo and AcdAdo were decomposed by UV light at wavelengths longer than 300 nm. Several products generated in AcdGuo and AcdAdo reactions were identified. All the identified products were previously reported as products caused by reactive oxygen species. Unlike the reaction of dCyd, products generated by the addition of a part of uric acid were not detected. Reportedly, several of these products generated in DNA induce mutation. If this DNA damage caused by uric acid with sunlight occurs in skin cells, mutations may arise. We should pay attention to the genotoxicity of uric acid in terms of DNA damage to dGuo and dAdo sites mediated by sunlight.

## Data Availability

Not applicable.

## Abbreviations

AcdGuo: 3′,5′-di-*O*-acetyl-2′-deoxyguanosine
AcdAdo: 3′,5′-di-*O*-acetyl-2′-deoxyadenosine
AcdSph: 3′,5′-di-*O*-acetyl derivative of spiroiminodihydantoin deoxyribonucleoside
AcdOz: 3′,5′-di-*O*-acetyl derivative of diamino-oxazolone deoxyribonucleoside
AcdIz: 3′,5′-di-*O*-acetyl derivative of amino-imidazolone deoxyribonucleoside
AcdIa^ox^: 3′,5′-di-*O*-acetyl derivative of dehydro-iminoallantoin deoxyribonucleoside
8-oxo-AcdGuo: 3′,5′-di-*O*-acetyl derivative of 7,8-dihydro-8-oxo-2′-deoxyguanosine
Ade: adenine
Fapy-AcdAdo: 3′,5′-di-*O*-acetyl derivative of formamidopyrimidine deoxyribonucleoside
5′,8-cyclo-AcdAdo: 3′,5′-di-*O*-acetyl derivative of 5′,8-cyclo-2′-deoxyadenosine
5′-deoxy-5′,8-cyclo-AcdAdo: 3′-*O*-acetyl derivative of 5′-deoxy-5′,8-cyclo-2′-deoxyadenosine
8-oxo-AcdAdo: 3′,5′-di-*O*-acetyl derivative of 7,8-dihydro-8-oxo-2′-deoxyadenosine

## References

[1] Wu XW, Lee CC, Muzny DM, Caskey CT (1989). Urate oxidase: primary structure and evolutionary implications. Proc Natl Acad Sci U S A.

[2] Oda M, Satta Y, Takenaka O, Takahata N (2002). Loss of urate oxidase activity in hominoids and its evolutionary implications. Mol Biol Evol.

[3] Ames BN, Cathcart R, Schwiers E, Hochstein P (1981). Uric acid provides an antioxidant defense in humans against oxidant- and radical-caused aging and cancer: a hypothesis. Proc Natl Acad Sci U S A.

[4] Sautin YY, Nakagawa T, Zharikov S, Johnson RJ (2007). Adverse effects of the classic antioxidant uric acid in adipocytes: NADPH oxidase-mediated oxidative/nitrosative stress. Am J Physiol Cell Physiol.

[5] Lanaspa MA, Sanchez-Lozada LG, Choi YJ, Cicerchi C, Kanbay M, Roncal-Jimenez CA, Ishimoto T, Li N, Marek G, Duranay M, Schreiner G, Rodriguez-Iturbe B, Nakagawa T, Kang DH, Sautin YY, Johnson RJ (2012). Uric acid induces hepatic steatosis by generation of mitochondrial oxidative stress: potential role in fructose-dependent and -independent fatty liver. J Biol Chem.

[6] Shi Y, Evans JE, Rock KL (2003). Molecular identification of a danger signal that alerts the immune system to dying cells. Nature..

[7] Hayashi N, Togawa K, Yanagisawa M, Hosogi J, Mimura D, Yamamoto Y (2003). Effect of sunlight exposure and aging on skin surface lipids and urate. Exp Dermatol.

[8] Yiu A, Van Hemelrijck M, Garmo H, Holmberg L, Malmström H, Lambe M, Hammar N, Walldius G, Jungner I, Wulaningsih W (2017). Circulating uric acid levels and subsequent development of cancer in 493,281 individuals: findings from the AMORIS study. Oncotarget..

[9] Suzuki T, Ozawa-Tamura A, Takeuchi M, Sasabe Y (2021). Uric acid as a photosensitizer in the reaction of deoxyribonucleosides with UV light of wavelength longer than 300 nm: identification of products from 2′-deoxycytidine. Chem Pharm Bull.

[10] Matsuda A, Shinozaki M, Suzuki M, Watanabe K, Miyasaka T (1985). Convenient method for the selective acylation of guanine nucleosides. Synthesis..

[11] Shibutani S, Takeshita M, Grollman AP (1991). Insertion of specific bases during DNA synthesis past the oxidation-damaged base 8-oxodG. Nature..

[12] Suzuki T, Nakamura A, Inukai M (2013). Reaction of 3′,5′-di-O-acetyl-2′-deoxyguansoine with hypobromous acid. Bioorg Med Chem.

[13] Luo W, Muller JG, Rachlin EM, Burrows CJ (2000). Characterization of spiroiminodihydantoin as a product of one-electron oxidation of 8-oxo-7,8-dihydroguanosine. Org Lett.

[14] Luo W, Muller JG, Rachlin EM, Burrows CJ (2001). Characterization of hydantoin products from one-electron oxidation of 8-oxo-7,8-dihydroguanosine in a nucleoside model. Chem Res Toxicol.

[15] Cadet J, Douki T, Ravanat J-L, Geacintov NE, Broyde S (2010). Oxidatively generated damage to isolated and cellular DNA. The chemical biology of DNA damage.

[16] Henderson PT, Delaney JC, Muller JG, Neeley WL, Tannenbaum SR, Burrows CJ, Essigmann JM (2003). The hydantoin lesions formed from oxidation of 7,8-dihydro-8-oxoguanine are potent sources of replication errors in vivo. Biochemistry..

[17] Raoul S, Berger M, Buchko GW, Joshi PC, Morin B, Weinfeld M, et al. ^1^H, ^13^C and ^15^N nuclear magnetic resonance analysis and chemical features of the two main radical oxidation products of 2′-deoxyguanosine: oxazolone and imidazolone nucleosides. J Chem Soc Perkin Trans 2. 1996:371–81.

[18] Duarte V, Gasparutto D, Jaquinod M, Cadet J (2000). In vitro DNA synthesis opposite oxazolone and repair of this DNA damage using modified oligonucleotides. Nucleic Acids Res.

[19] Chattopadhyaya R, Goswami B (2012). Oxidative damage to DNA constituents by iron-mediated Fenton reactions: the deoxyadenosine family. J Biomol Struct Dyn.

[20] Vieira AJSC, Steenken S (1990). Pattern of hydroxy radical reaction with adenine and its nucleosides and nucleotides. Characterization of two types of isomeric hydroxy adduct and their unimolecular transformation reactions. J Am Chem Soc.

[21] Delaney MO, Wiederholt CJ, Greenberg MM (2002). Fapy-dA induces nucleotide misincorporation translesionally by a DNA polymerase. Angew Chem Int Ed Engl.

[22] Koag M-C, Jung H, Lee S (2020). Mutagenesis mechanism of the major oxidative adenine lesion 7,8-dihydro-8-oxoadenine. Nucleic Acids Res.

[23] Balasubramanian B, Pogozelski WK, Tullius TD (1998). DNA strand breaking by the hydroxyl radical is governed by the accessible surface areas of the hydrogen atoms of the DNA backbone. Proc Natl Acad Sci U S A.

[24] Dizdaroglu M, Jaruga P (2012). Mechanisms of free radical-induced damage to DNA. Free Radic Res.

[25] Locatelli GA, Pospiech H, Tanguy Le Gac N, van Loon B, Hubscher U, Parkkinen S, Syväoja JE, Villani G (2010). Effect of 8-oxo-guanine and abasic site DNA lesions on in vitro elongation by human DNA polymerase ϵ in the presence of replication protein a and proliferating cell nuclear antigen. Biochem J.

[26] Kamiya H, Suzuki M, Ohtsuka E (1993). Mutation-spectrum of a true abasic site in codon 12 of a c-ha-ras gene in mammalian cells. FEBS Lett.

[27] Cabral Neto JB, Cabral RE, Margot A, Le Page F, Sarasin A, Gentil A (1994). Coding properties of a unique apurinic/apyrimidinic site replicated in mammalian cells. J Mol Biol.

[28] Cadet J, Vigny P, Morrison H (1990). The photochemistry of nucleic acids, chap. 1. Bioorganic Photochemistry: Photochemistry and the Nucleic Acids.

[29] von Sonntag C (2006). Free-radical-induced DNA damage and its repair: a chemical perspective.

